# The Proinflammatory Role of Guanylate-Binding Protein 5 in Inflammatory Bowel Diseases

**DOI:** 10.3389/fmicb.2022.926915

**Published:** 2022-06-02

**Authors:** Yichen Li, Xutao Lin, Wenxia Wang, Wenyu Wang, Sijing Cheng, Yibo Huang, Yifeng Zou, Jia Ke, Lixin Zhu

**Affiliations:** ^1^Guangdong Provincial Key Laboratory of Colorectal and Pelvic Floor Diseases, Department of Colorectal Surgery, The Sixth Affiliated Hospital, Guangdong Institute of Gastroenterology, Sun Yat-sen University, Guangzhou, China; ^2^Guangdong Provincial Key Laboratory of Colorectal and Pelvic Floor Diseases, Department of Gastrointestinal Endoscopy, The Sixth Affiliated Hospital, Guangdong Institute of Gastroenterology, Sun Yat-sen University, Guangzhou, China; ^3^School of Medicine, Sun Yat-sen University, Shenzhen, China

**Keywords:** Crohn’s disease, ulcerative colitis, inflammasome, pyroptosis, guanylate binding protein

## Abstract

NLRP3 inflammasome is implicated in the pathogenesis of inflammatory bowel diseases (IBD). Since guanylate-binding protein 5 (GBP5) induces the NLRP3 inflammasome activity, we aim to investigate the potential role of GBP5 in IBD pathogenesis. The expression of GBP5, NLRP3 inflammasome, and related cytokines and chemokines was examined in two cohorts of IBD patients and healthy controls, by microarray transcriptome analysis and quantitative real-time PCR. Cellular localization of GBP5 in colonic biopsies was examined by immunohistochemistry and immunofluorescence with confocal microscopy. For functional studies, *GBP5* was induced by interferon γ or silenced by siRNA or CRISPR/CAS9 technique, and inflammatory activities were evaluated at mRNA and protein levels. We found that the expression of *GBP5* was elevated in colonic mucosa in two geographically and culturally distinct IBD cohorts. In colonic tissues of IBD patients, GBP5 expression was mainly confined to immune cells and the levels of *GBP5* expression were correlated with those of the inflammatory cytokines and chemokines. In cultured T and macrophage cells, the expression of proinflammatory cytokines and chemokines was increased when *GBP5* was induced, while *GBP5* deficiency leads to decreased expression of proinflammatory mediators including gasdermin D, caspase 1, cytokines, and chemokines. We conclude that GBP5 is required in the expression of many proinflammatory cytokines and chemokines in intestinal immune cells. In addition, GBP5 may upregulate inflammatory reactions through an inflammasome-mediated mechanism. Since GBP5 plays a proinflammatory role at the early steps of the inflammatory cascades of IBD pathogenesis, and is implicated in IBD patients of distinct genetic and environmental backgrounds, targeting GBP5 could be an effective strategy for the management of IBD.

## Introduction

Inflammatory bowel diseases (IBD), including Crohn’s disease (CD) and ulcerative colitis (UC), are chronic and relapsing inflammatory diseases mainly affecting the intestines. The pathogenesis of IBD is not known, but generally believed to be driven by abnormalities in genetics, environment, gut microbiota, and immunity (Seyed Tabib et al., 2020).

NLRP3 (NOD-like receptor family pyrin domain containing 3) inflammasome comprises the inflammasome sensor NLRP3, the adaptor protein ASC (apoptosis-associated speck-like protein containing a CARD domain), and the effector caspase 1. Activation of NLRP3 inflammasome leads to the production of active form of proinflammatory cytokines IL1β and IL-18. Different groups have reported that colonic mucosa of IBD patients exhibited higher levels of caspase 1 (McAlindon et al., 1998), IL1β (Mahida et al., 1989; Ligumsky et al., 1990; Reinecker et al., 1993; McAlindon et al., 1998), and IL-18 (Kanai et al., 2000), demonstrating an association of elevated NLRP3 inflammasome activity with IBD. Animal studies provided a chain of evidence in support of a key role for NLRP3 inflammasome in colitis. NLRP3 gene knockout mice are protected from dextran sodium sulfate (DSS) (Bauer et al., 2010) or 2,4,6-trinitrobenzene sulfonic acid (TNBS) (Bauer et al., 2012) induced colitis compared to wild-type animals. Similar protection was also observed with pharmacological inhibition of caspase 1 with pralnacasan (Bauer et al., 2010) or with caspase 1 knockout mice (Siegmund et al., 2001; Blazejewski et al., 2017). Along this line, inhibition of IL1β or its receptor suppressed experimental colitis (Seo et al., 2015; Neudecker et al., 2017). Importantly, IL-1 receptor blockade using anakinra resulted in a rapid and sustained improvement in patients with colitis (de Luca et al., 2014).

Further support for a causal role of NLRP3 inflammasome in IBD pathogenesis came from studies on CARD8, an inhibitor for NLRP3. Single-nucleotide polymorphism (SNP) studies found that a loss-of-function mutation in CARD8 is associated with IBD (Fisher et al., 2007; Yang et al., 2011). Without the inhibitory CARD8 activity, the unchecked NLRP3 inflammasome activity leads to elevated IL1β and consequently intestinal inflammation. Patients with CARD8 loss-of-function mutation specifically responded to IL1β blockers, but not to anti-TNFα (Mao et al., 2018), demonstrating the role of CARD8-NLRP3-IL1β axis in IBD.

Guanylate-binding protein 5 (GBP5), a member of the GBP family, is a GTPase highly inducible by interferon (Cheng et al., 1983). GBP5 has an emerging role in mediating cell autonomous defenses against intracellular pathogens including those of *Francisella novicida* (Meunier et al., 2015), *Toxoplasma gondii* (Matta et al., 2018), and HIV-1 (Krapp et al., 2016). Peripheral blood mononuclear cells (PBMCs) from children with decreased GBP5 expression were more susceptible to respiratory syncytial virus compared to adult (Li et al., 2020). Under pathological conditions, abnormal upregulated expression of GBP5 is caused by dysregulated immune responses, such as rheumatoid arthritis-affected synovial tissue (Haque et al., 2021) and some human malignancies including medullary carcinoma (Friedman et al., 2016), gastric adenocarcinomas (Patil et al., 2018), and glioblastoma (Yu et al., 2021). GBP5, but not other members of the GBP family, promotes the assembly of the NLRP3 inflammasome in response to live *L. monocytogenes*, *S. Typhimurium*, and soluble inflammasome priming agents, as demonstrated in *GBP5* knockout mice and cell culture models (Shenoy et al., 2012; Santos et al., 2018). Consistent with its effect on inflammasome assembly, GBP5 was reported to stimulate NF-κB signaling, induce the expression of interferon (IFN) and other proinflammatory factors, and inhibit the replication of influenza A virus in a cell culture model (Feng et al., 2017). The dysregulation of the immune–microbiome axis is an important cause of IBD (Graham and Xavier, 2020). In the presence of microbial ligands, NLRP3 inflammasome is crucial for regulation of intestinal homeostasis (Tourkochristou et al., 2019). Therefore, we hypothesize that GBP5 plays a key role in IBD by regulating NLRP3 inflammasome activity. Here, we show that GBP5 is highly elevated in the colonic mucosa of IBD patients and that GBP5 is required in the stimulated secretion of proinflammatory mediators in cell culture models.

## Results

### Elevated Guanylate-Binding Protein 5 Expression in the Colonic Mucosa of Inflammatory Bowel Diseases Patients

**FIGURE 1:**
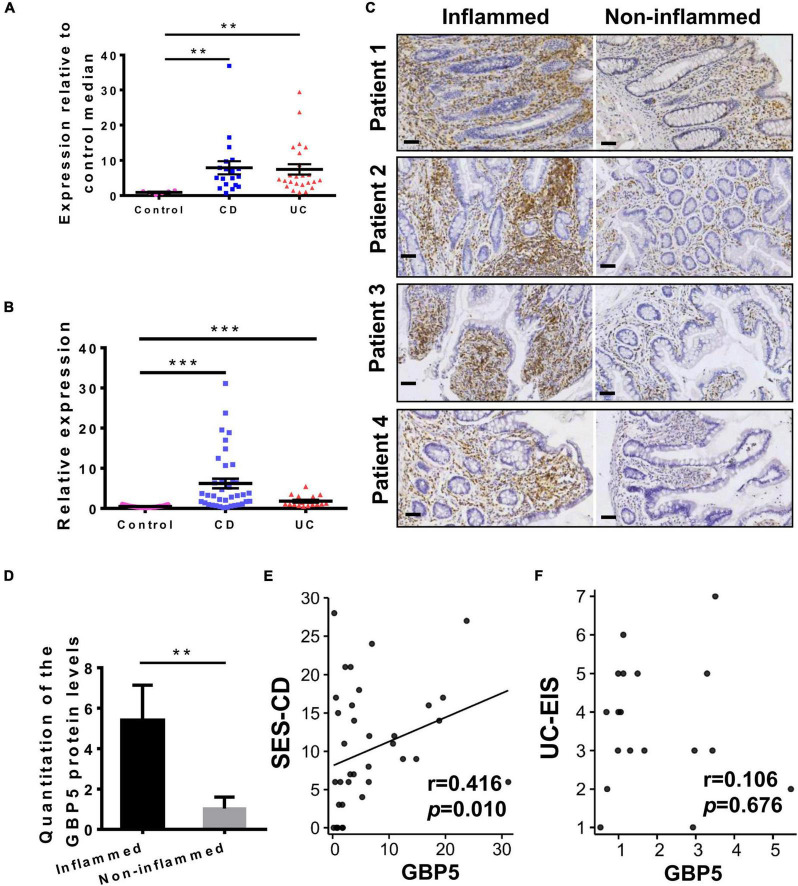
GBP5 is highly expressed in the inflamed intestinal tissue of IBD patients. **(A)** Messenger RNA expression of GBP5 in the colonic mucosa of healthy controls (*n* = 6), patients with Crohn’s disease (CD, *n* = 19), and ulcerative colitis (UC, *n* = 24). Data are from a microarray dataset generated from a European cohort. **P < 0.01, Dunn’s multiple comparison test. **(B)** Quantitative RT-PCR analysis of GBP5 mRNA in colonic mucosa from a Chinese cohort including healthy controls (*n* = 35), and patients with CD (*n* = 38) and UC (*n* = 17). ***P < 0.001, Dunn’s multiple comparison test. **(C)** Immunohistochemical staining of GBP5 in colonic mucosa from four representative patients with CD. Images of inflamed and non-inflamed sites from the same patient are compared side by side. Bar = 50 μm. **(D)** Quantitation of the GBP5 staining in the inflamed and non-inflamed sites in **(C)**. **P < 0.01, paired Student’s *t*-test. **(E,F)** Spearman’s correlation analysis of the mucosal GBP5 expression levels and endoscopic severities. GBP5 expression levels are from quantitative RT-PCR results. SES-CD, simple endoscopic score for Crohn’s disease; UC-EIS, ulcerative colitis endoscopic index of severity.

We first examined *GBP5* mRNA expression in patients with IBD using a published transcriptome dataset generated with colonic biopsies from European patients. The demographic and clinical characteristics of these patients were described previously (Arijs et al., 2009). Elevated *GBP5* expression was observed in both CD and UC patients compared to healthy controls, with fold changes of 8.6 and 8.1, respectively (Figure 1A). To confirm these findings, a validation cohort including healthy controls, CD, and UC patients from south China was enrolled (Supplementary Table 1) and *GBP5* mRNA levels in colonic mucosa were evaluated by quantitative real-time PCR (qRT-PCR). Similarly, elevated *GBP5* expression was observed in CD and UC patients compared to healthy controls, with fold changes of 13.1 and 3.8, respectively (Figure 1B).

To examine the GBP5 expression at protein level, inflamed and adjacent non-inflamed colonic tissues from the same CD patients who underwent colon resection were subjected to immunohistochemistry (IHC) staining. With all the samples examined, GBP5 staining was more intense in the inflamed tissue than in the non-inflamed tissue (Figures 1C,D). Interestingly, GBP5 positive cells were mostly found in the lamina propria, with a few exceptions located at the luminal or glandular epithelium (Figure 1C).

To understand the potential role of GBP5 in clinical outcome, correlation analysis between mucosal GBP5 expression and disease degree of IBD was performed. We used the simple endoscopic score for Crohn’s disease (SES-CD) and the ulcerative colitis endoscopic index of severity (UC-EIS) to evaluate the severity of CD and UC, respectively. The expression of GBP5 was positively correlated with SES-CD (Figure 1E, Spearman’s correlation coefficient = 0.416; *P* = 0.010), but not correlated with UC-EIS (Figure 1F, Spearman’s correlation coefficient = 0.106; *P* = 0.676).

### Immune Cell-Specific Guanylate-Binding Protein 5 Expression in the Colon of Crohn’s Disease Patients

**FIGURE 2:**
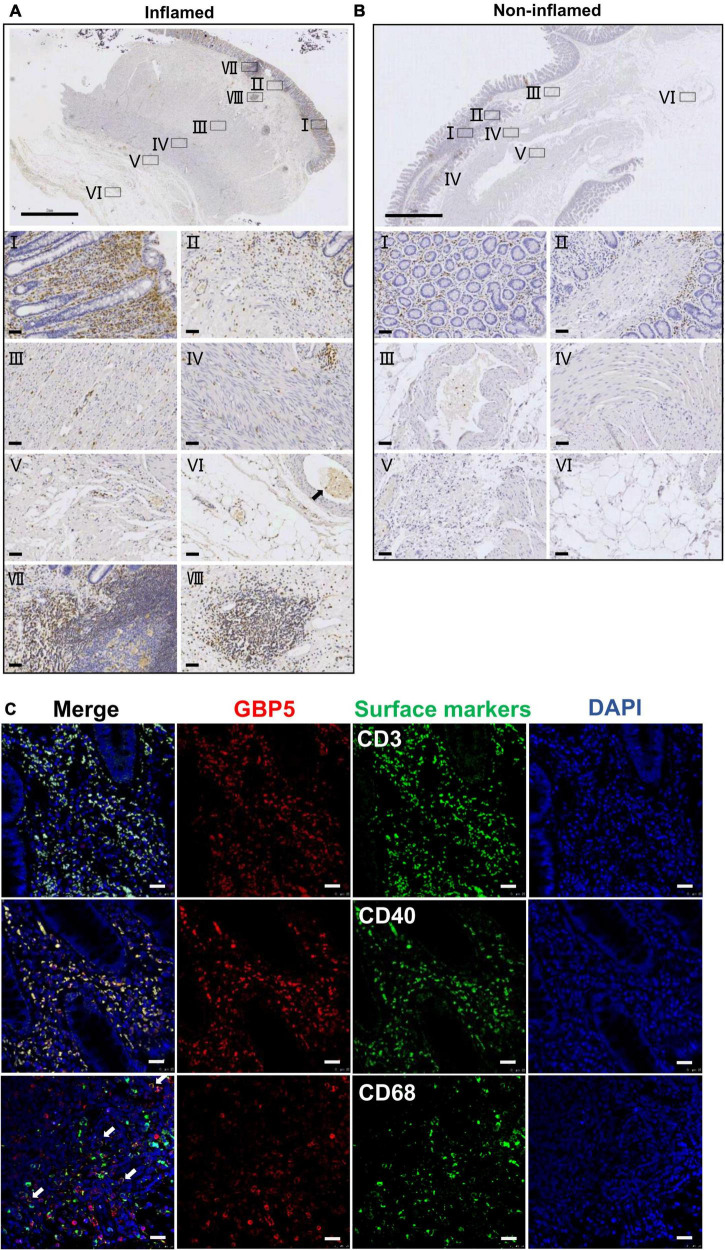
Immune cell-specific GBP5 expression in patients with CD. **(A)** Immunohistochemical staining of a colonic biopsy section from a representative CD patient with an anti-GBP5 antibody: an inflamed area. The top image shows a panoramic view of the section. Bar = 2 mm. Details are shown for boxed areas representing different anatomic structures. I, mucosa; II, muscularis mucosa and submucosa; III, circular muscle; IV, longitudinal muscle; V, serosa; VI, mesentery, black arrow indicates a blood vessel; VII, Peyer’s patch in mucosa; VIII, lymph node in submucosa. Bar = 50μm. **(B)** Immunohistochemical staining of a colonic biopsy section from a representative CD patient with an anti-GBP5 antibody: a non-inflamed area. The top image shows a panoramic view of the section. Bar = 2 mm. Details are shown for boxed areas representing different anatomic structures. I, mucosa; II, lamina propria, muscularis mucosa and submucosa; III, a blood vessel in submucosae layer; IV, circular muscle and longitudinal muscle; V, serosa; VI, mesentery. Bar = 50μm. **(C)** Immunofluorescence staining of inflamed colon tissue from a representative CD patient with antibodies against GBP5, CD3, CD40, and CD68. White arrow indicates the overlap of GBP5 and CD68. Bar = 50 μm.

To better understand the tissue distribution of GBP5, IHC staining of GBP5 was examined at the entire depth of the inflamed and non-inflamed colonic tissues of CD patients who underwent colon resection. In the inflamed colon, GBP5 positive cells were densely populated in mucosa, mostly lamina propria (Figure 2AI). Besides, GBP5 positive cells were frequently observed in other layers of the colonic tissue including submucosa, circular muscle, longitudinal muscle, serosa, and mesentery (Figures 2AII–VI). Less GBP5 positive cells were observed in colonic layers of the non-inflamed tissue from the same patient (Figure 2B). It is noteworthy that GBP5 was not detected in muscle cell (Figures 2AIII,IV) or endothelial cell, but detected in the blood cells (Figure 2AVI). Another outstanding observation was the enrichment of GBP5 positive cells in Peyer’s patch (mucosa, Figure 2AVII) and lymph node (submucosa, Figure 2AVIII). The elevated expression of GBP5 across the colonic layers of CD patients is in line with the fact that CD is usually inflicted by transmural inflammation.

To determine the cellular distribution of GBP5, inflamed colon tissues from CD patients were subjected to immunofluorescence staining for GBP5 and immune cell marker proteins. Confocal microscopy showed that most of the CD3 positive cells, most of the CD40 positive cells, and the majority of CD68 positive cells expressed GBP5 (Figure 2C). CD3, CD40, and CD68 are marker proteins for T lymphocytes, antigen-presenting cells (dendritic cells, macrophages, and B cells) (Croft and Siegel, 2017), and macrophages, respectively. Thus, the above results demonstrated immune cell-specific expression of GBP5.

### Transcriptome Analysis Reveals Association Between Guanylate-Binding Protein 5 and Inflammatory Reaction Pathways

**FIGURE 3:**
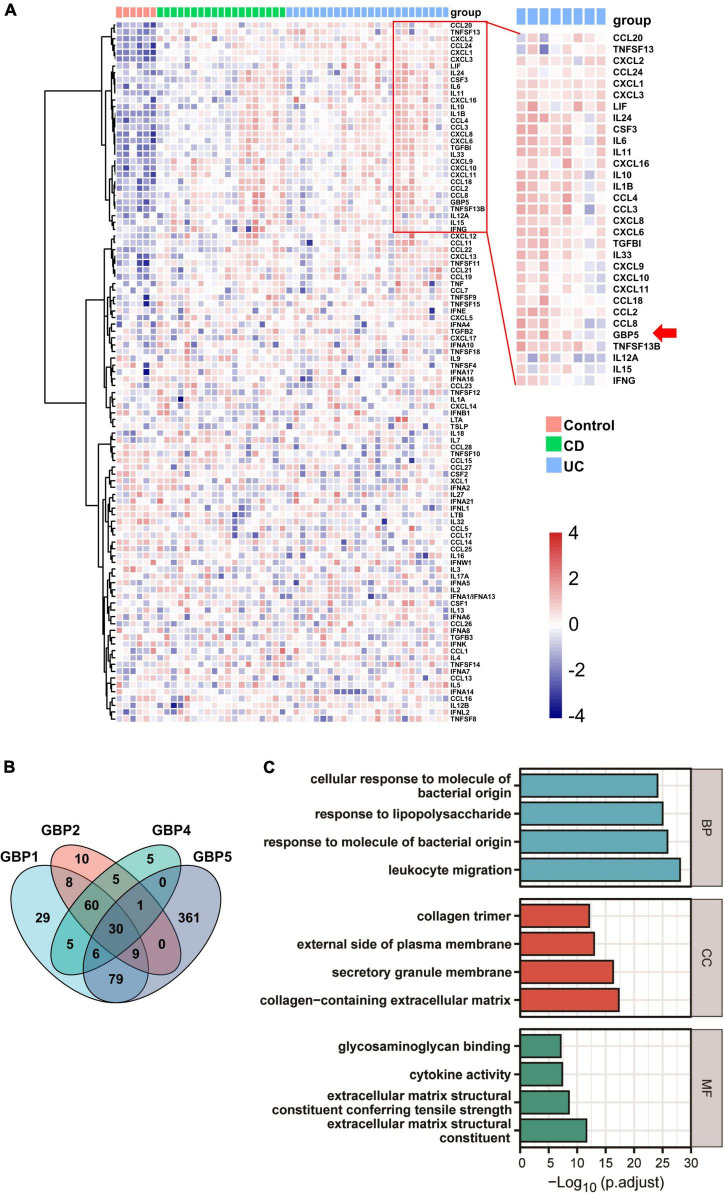
GBP5 is implicated in inflammatory processes in IBD according to transcriptome analysis. **(A)** Heatmap for mRNA expression levels of GBP5 (arrow), available cytokines, and chemokines based on transcriptome data generated from colonic mucosal biopsies of healthy controls (*n* = 6), patients with Crohn’s disease (CD, *n* = 19), and ulcerative colitis (UC, *n* = 24). Unsupervised hierarchical clustering of *GBP5*, cytokine, and chemokine genes was performed. Genes in the red box are more closely clustered with GBP5. **(B)** Venn plot of the number of genes associated with GBP1, GBP2, GBP4, and GBP5, based on their mRNA expression levels. Associations with Pearson’s correlation coefficient no less than 0.6 are counted. **(C)** Gene Ontology (GO) analysis of GBP5 associated genes. The cutoff value for the input gene list is Pearson’s correlation coefficient no less than 0.6. BP, biological process; CC, cellular compartment; MF, molecular function. All samples from GSE16879 are included (*n* = 90).

To identify potential links between *GBP5* and IBD pathogenesis, we performed hierarchical clustering of *GBP5* with 102 available cytokine and chemokine genes with the transcriptome dataset generated from colonic mucosa of IBD patients (GSE16879) (Arijs et al., 2009). The clustering result showed that *GBP5* shared a similar expression pattern with gene coding for proinflammatory cytokines and chemokines including *IL1B* and *IL-6*, and they were highly elevated in most of the CD and UC patients compared to the healthy controls (Figure 3A). Interestingly, the anti-inflammatory cytokine *IL-10* exhibited a similar expression pattern as *GBP5*. The elevated *IL-10* expression in IBD was observed previously and was thought to reflect a futile effort of patient immune system to control the excessive inflammatory reaction (Autschbach et al., 1998). Next, we performed Pearson’s correlation analyses between every two genes based on the transcriptome data (Figure 3B). With a threshold of correlation coefficient greater than 0.6, 486 genes were correlated with *GBP5*. In comparison, 226, 123, and 112 genes were correlated with *GBP1*, *GBP2*, and *GBP4*, respectively. Apparently, at the transcription level, *GBP5* had the largest impact on IBD among all *GBP* family genes. We then performed Gene Ontology (GO) enrichment analysis with all 486 genes correlated with *GBP5*. The top “biological process,” “cellular compartment,” and “molecular function” are listed in Figure 3C. The top “biological process,” including “leukocyte migration,” “response to molecule of bacterial origin,” “response to lipopolysaccharide,” and “cellular response to molecule of bacterial origin,” is related to inflammatory responses to bacterial infection. In addition, the top “cellular compartment” and the top “molecular function,” such as “collagen-containing extracellular matrix” and “extracellular matrix structural constituent,” are closely related to lymphocyte migration and infiltration. These results are consistent with previous reports that GBP5 plays a critical role in host defense against bacterial pathogens (Meunier et al., 2014; Pilla et al., 2014).

### Guanylate-Binding Protein 5 Deficiency Downregulates Proinflammatory Chemokines and Cytokines in Cultured Cells

For further understanding of the association between *GBP5* and inflammatory processes, we performed *GBP5* siRNA knockdown in Jurkat cells to determine the impact of *GBP5* on chemokine and cytokine secretion. Diminished GBP5 expression in Western blot analysis indicated efficient knockdown of *GBP5* gene in Jurkat cells (Supplementary Figure 1A). The cell culture supernatants were then subjected to Luminex chemokine and cytokine assay. Compared to cells treated with control RNA, upon stimulation with IFNγ and lipopolysaccharide (LPS), *GBP5* siRNA-treated cells exhibited decreased levels of CCL2, CCL8, CCL13, CCL25, CXCL10, CXCL11, CXCL12, CXCL16, CX3CL1, IL-1β, IL-10, and MIF in the cell culture supernatant (Supplementary Figure 1B). In addition, CCL19, CCL20, CCL22, CXCL2, and CXCL13 were not detected in the supernatant after *GBP5* knockdown, but detected in the supernatant of the cells treated with control RNA (Supplementary Figure 1B). *GBP5* knockdown also caused a trend of decreased levels in some proinflammatory chemokines and cytokines, including CCL1, CCL11, CCL15, CCL21, CCL26, CCL27, CXCL1, CXCL5, IL-16, and TNF-α, but statistical significance was not achieved (Supplementary Figure 1B). CCL7, CCL17, CCL23, CXCL6, CXCL9, GM-CSF, IL-2, IL-4, and IL-6 were not detected in any of the samples (Supplementary Figure 1B).

**FIGURE 4:**
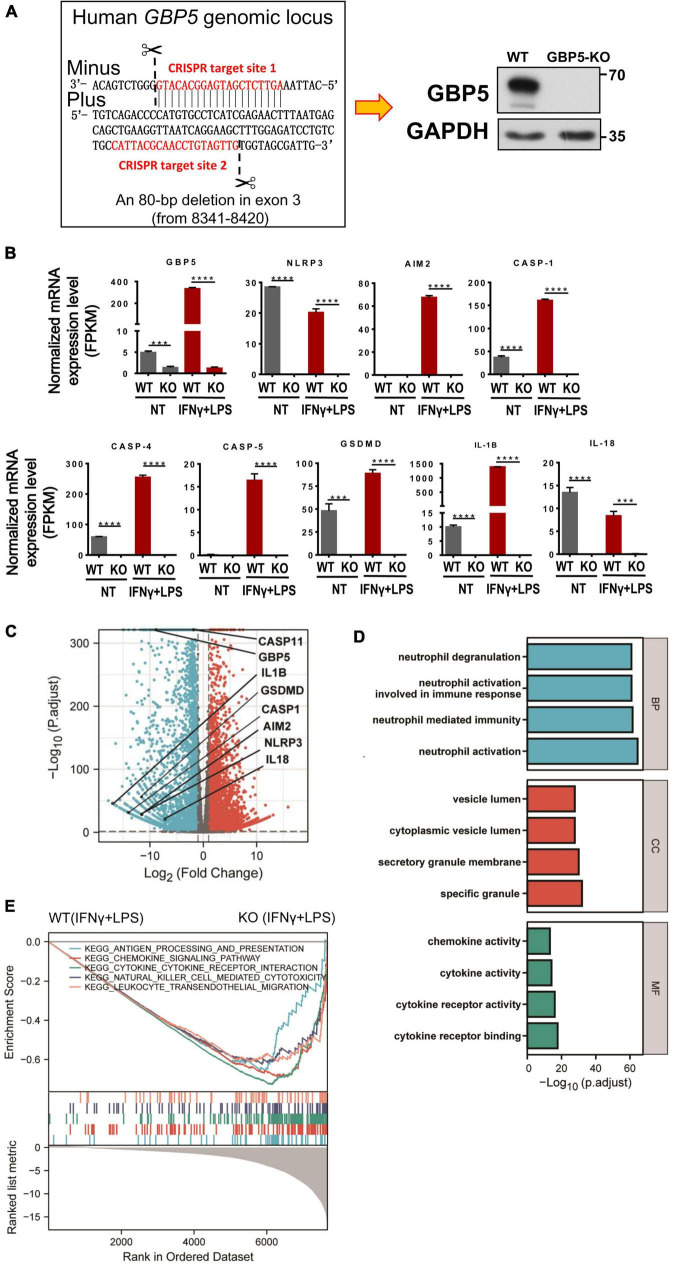
*GBP5* knockout downregulated the expression of proinflammatory mediator genes in THP-1 cells. The transcriptomes of wild-type and *GBP5^–/–^* THP-1 cells, with or without stimulation [IFNγ and lipopolysaccharide (LPS)], respectively, were analyzed by RNAseq technique. N = 3 for each treatment group. **(A)** Generation of *GBP5* knockout (*GBP5^–/–^*). THP-1 cell line by CRISPR/Cas9-mediated genome editing. The two CRISPR target sites are highlighted in red. Loss of GBP5 was confirmed by Western blot. WT, wild-type control. **(B)** Messenger RNA expression of *GBP5* and related genes in wild-type and *GBP5^–/–^* THP-1 cells (clone B2), with or without stimulation (IFNγ and LPS), respectively. FPKM, fragments per kilobase million. ***P < 0.001; ****P < 0.0001; unpaired Student’s *t*-test. **(C)** Differential gene expression between wild-type and *GBP5^–/–^* THP-1 cells: volcano plot. Vertical and horizontal dashed lines indicate the cutoff values for differentially expressed genes:| Log_2_ (Fold Change)| > 1 and p.adjust < 0.05. **(D)** GO (Gene Ontology) analysis of downregulated genes in *GBP5* knockout cells. The cutoff values for selecting differentially expressed genes for the input gene list: Log_2_(Fold Change) < -2, and p. adjust < 0.05. BP, biological process; CC, cellular compartment; MF, molecular function. **(E)** Gene set enrichment analysis (GSEA) of *GBP5^–/–^* THP-1 cells (clone B2) transcriptome compared to wild-type THP-1 cells. Upper panel: the enrichment score curves of the top KEGG (Kyoto Encyclopedia of Genes and Genomes) pathways exhibiting decreased expression in *GBP5^–/–^* THP-1 cells: immune related pathways. Middle panel: distribution of the genes related to the pathways indicated in the upper panel. The genes were ranked according to their differential expression between wild-type and *GBP5^–/–^* THP-1 cells. Genes of higher rank (left) exhibit relatively higher expression in wild-type THP-1 cells. Lower panel: Graphical representation of the correlations of the gene expression levels with the phenotypes: wild-type or *GBP5* knockout. Genes on the right are more negatively correlated with *GBP5* deficiency.

For a precise evaluation of *GBP5* impact on chemokine and cytokine secretion, *GBP5* gene was removed from THP-1 cell by CRISPR/CAS9 method and confirmed by Western blot showing no GBP5 expression in *GBP5^–/–^* THP-1 cells (Figure 4A). Global mRNA expression of the *GBP5^–/–^* THP-1 cells was assessed by RNA sequencing. As expected, *GBP5* mRNA was reduced in *GBP5^–/–^* THP-1 cells compared to the wild-type controls, with or without induction by IFNγ plus LPS (Figures 4B,C). Surprisingly, *GBP5* deficiency greatly reduced the mRNA expressions of many inflammation and immune related genes, including (1) IFNγ response genes such as *AIM2, CASP1, GSDMD*, and *IL1B*, and (2) IFNγ non-response genes such as *NLRP3, CASP4, CASP5*, and *IL-18* (Figure 4B).

Comparing the *GBP5^–/–^* with wild-type THP-1 cells, many differentially expressed genes, including 2,298 genes upregulated and 2,813 genes downregulated in *GBP5^–/–^* cells, were identified (Figure 4C). The mRNA expression for most proinflammatory cytokines and chemokines was reduced or undetected in *GBP5^–/–^* cells (Supplementary Figure 2). We then performed GO enrichment analysis with the list of genes downregulated in *GBP5^–/–^* cells. The identified top “biological process,” “cellular compartment,” and “molecular function” are related to inflammatory signaling, immune cell migration, neutrophil activation, secretion of cytokines and chemokines, and other immune and inflammatory events (Figure 4D).

Using a different approach for functional analysis, gene set enrichment analysis (GSEA) identified a similarly broad suppression of immune and inflammatory functions in *GBP5^–/–^* THP-1 cells, including antigen processing, chemokine signaling, cytokine signaling, leukocyte migration, and NK cell cytotoxicity (Figure 4E). Compared to GO enrichment analysis that uses partial information of the biological pathways (in our case, the downregulated genes), GSEA considers all available information of relevant genes for functional analysis. Thus, both methods identified immune and inflammatory pathways as downregulated pathways in *GBP5^–/–^* THP-1 cells.

**FIGURE 5:**
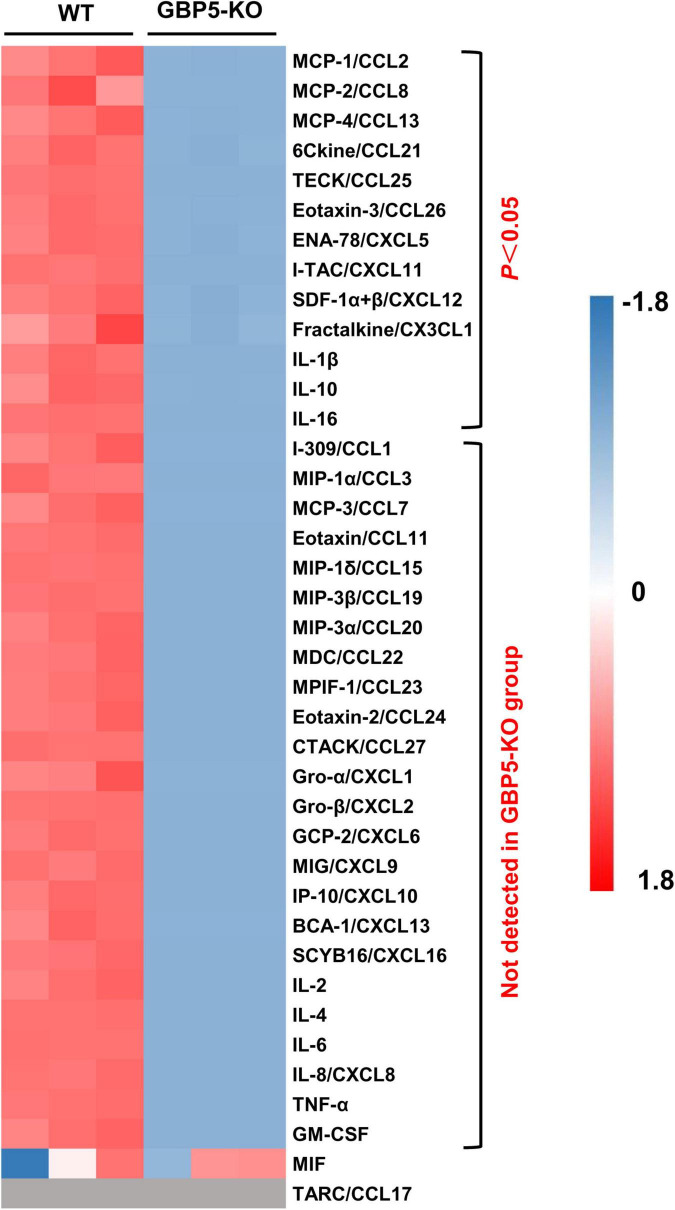
Decreased chemokine and cytokine secretion in *GBP5* knockout THP-1 cells. The protein levels of chemokines and cytokines in the cell culture supernatant of wild-type (WT) and *GBP5* knockout (KO) THP-1 cells are plotted. Cells were primed with IFNγ and LPS before sample collection. The concentrations of cytokines and chemokines were determined by Luminex liquid suspension chip. Data were normalized as (x-mean)/SD. Gray blocks indicate no detection. *P*-values were from Student’s *t*-tests.

Next, we examined the altered immune and inflammatory pathways at protein level. Compared to the wild-type controls, THP-1 cells with *GBP5* deficiency exhibited decreased levels of CCL2, CCL8, CCL13, CCL21, CCL25, CCL26, CXCL5, CXCL11, CXCL12, CX3CL1, IL1β, IL-10, and IL-16 in the cell culture supernatant (Figure 5). In addition, CCL1, CCL3, CCL7, CCL11, CCL15, CCL19, CCL20, CCL22, CCL23, CCL24, CCL27, CXCL1, CXCL2, CXCL6, CXCL9, CXCL10, CXCL13, CXCL16, IL-2, IL-4, IL-6, CXCL8, TNF-α, and GM-CSF were not detected in the supernatant of *GBP5* knockout cells, but detected in the WT controls (Figure 5). CCL17 was again not detected in any of the samples (Figure 5). Therefore, *GBP5* deficiency led to decreased secretion for most of the inflammatory mediators analyzed.

**FIGURE 6:**
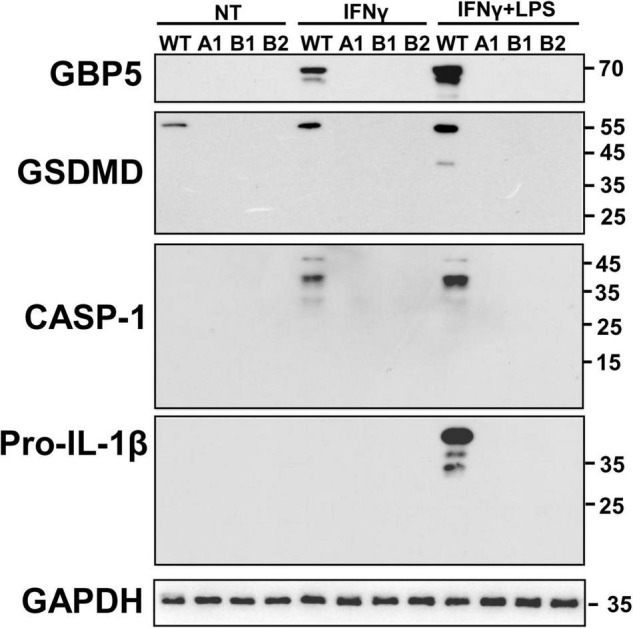
Diminished expression of inflammasome related proteins in *GBP5* knockout THP-1 cells was subjected to wild-type THP-1 cells, and *GBP5* knockout THP-1 cell clones (A1, B1, and B2) were left untreated (NT), treated with IFNγ (25 ng/ml) only, or treated with IFNγ (25 ng/ml) plus LPS (500 ng/ml) for 16 h, before Western blot analysis with antibodies against GBP5, gasdermin D (GSDMD), caspase 1 (CASP1), pro-IL1β, and GAPDH, respectively.

Given the close relation among GBP5, inflammasomes, and IL1β, we examined the intracellular protein expression of some related molecules. Western blots showed that *GBP5* deficiency greatly decreased the expression of gasdermin D, caspase 1, and pro-IL1β (Figure 6). Since the active form for gasdermin D or caspase 1 was not observed in any of the samples, the decreased production of IL1β is not likely due to impaired proteolytic activation by inflammasomes in *GBP5* deficiency. Rather, the transcription and expression analyses indicate that the decreased IL1β production is part of the consequence of broad inhibition of inflammatory gene expression in *GBP5* deficiency.

## Discussion

Here, we show for the first time the elevated expression of *GBP5* in colonic mucosa of patients with IBD and that *GBP5* is required for the stimulated secretion of inflammatory cytokines and chemokines, including IL1β, from T lymphocytes and macrophages. In colonic tissues of IBD patients, the expression of GBP5 was mainly confined to immune cells and the levels of *GBP5* expression were correlated with those of the inflammatory markers. Importantly, the expression of proinflammatory cytokines and chemokines was increased when *GBP5* was induced, while *GBP5* deficiency leads to decreased production of the proinflammatory mediators in T and macrophage cells. Thus, the specific role of GBP5 in IBD pathogenesis is to facilitate the stimulated production of inflammatory mediators in intestinal immune cells. Our observation of highly elevated *GBP5* expression at the inflamed colonic mucosa in two geographically and culturally distinct IBD cohorts indicates that GBP5 plays a common and important role in the early steps of IBD pathogenesis and, therefore, is a potential therapeutic target for the management of IBD patients of various genetic and environmental backgrounds.

Our results suggest that GBP5 may promote inflammation through two mechanisms. First, gene knockout studies indicated that *GBP5* is required for the stimulated expression of many inflammatory cytokines, such as IL1β, and is required for the expression of many IFNγ non-response cytokines, such as IL-18. Studies at both mRNA and protein levels indicated that GBP5 had a large impact on the expression of these proinflammatory cytokines and chemokines. Once IL1β is induced, in turn, IL1β may stimulate the production of other proinflammatory cytokines and chemokines including IL-6, IL8, CCL2, CCL5, CXCL1, CXCL2, CXCL3, CXCL6, and IFNγ (Cooper et al., 2001; Hengartner et al., 2015). The outstanding knowledge gap is how the cytoplasmic inflammasome interacting GBP5 regulates gene expression? Second, since GBP5 promotes NLRP3 inflammasome activity (Shenoy et al., 2012), and NLRP3 inflammasome is required for the excessive inflammatory reactions in IBD (reviewed in Yuan et al., 2018), it is expected that NLRP3 inflammasome may mediate the proinflammatory effect of GBP5. This is supported by our observation that the expression of caspase 1, gasdermin D, and IL1β was elevated when *GBP5* is induced and was reduced when *GBP5* was downregulated by siRNA or gene knockout. The puzzle here is the observation that the active forms of caspase 1 and gasdermin D were not observed in IFNγ primed THP-1 cells. For both mechanisms, future efforts are needed to unravel the puzzles and to join the knowledge gaps.

One major challenge for the interpretation of our data came from a few studies that reported protective role for NLRP3 inflammasome in mouse models of colitis, that is, mice lacking NLRP3, ASC, or caspase 1 were more susceptible for colitis in DSS and azoxymethane (AOM) models (Allen et al., 2010; Dupaul-Chicoine et al., 2010; Zaki et al., 2010a,b; Hirota et al., 2011). The discrepancies regarding the role of NLRP3 inflammasome in colitis may be explained by environmental factors including microbiota (Bauer et al., 2012) and diet (Youm et al., 2015). In addition, Zaki et al. (2010a) argued that the protective role of NLRP3 inflammasome is due to its beneficial effect on epithelial barrier function via induction of IL-18 in epithelial cells. However, this argument does not reconcile with the facts that little NLRP3 inflammasome activity presents in the intestinal epithelial cells and that IL-18 production by these cells is independent of NLRP3 inflammasome (Yao et al., 2017).

Besides the conventional NLRP3 inflammasome-mediated mechanism, a caspase-11-dependent pathway may relay the inflammatory signal from GBP5. Mice and cell culture studies suggested that, upon detection of cytoplasmic LPS, GBP proteins encoded on mouse chromosome 3 activate caspase-11-dependent cell autonomous immune responses (Pilla et al., 2014). This is consistent with current knowledge that structural and functional alterations in the gut microbiota, especially in gram-negative bacteria (Palmela et al., 2018), and their cell wall component LPS (Caradonna et al., 2000), are implicated in the pathogenesis of IBD. In support of a causal role for bacteria and LPS in IBD, we identified the following pathways as the top features of the GBP5 associated colonic mucosal transcriptome in IBD: “response to molecule of bacterial origin,” “response to lipopolysaccharide,” and “cellular response to molecule of bacterial origin.” On the contrary, it is noteworthy that virus infection is implicated in IBD pathogenesis (Yang et al., 2019), and GBP5 has been identified as an IFN-induced virus restriction factor that interferes with virus assembly (Krapp et al., 2016), or through induction of innate immune mediators (Feng et al., 2017). Further study is required to clarify potential collaborative roles of caspase-11 and GBP5 in IBD pathogenesis, and whether viral or bacterial infection triggers elevated GBP5 expression in the intestines of IBD.

The limitations of our study include lack of *in vivo* study and small sample size. Therefore, further experimentations have been planned to validate our findings. In addition, other interesting questions emerged from our study. For example, GBP5 seemed to play a more important role in CD than in UC. With the prospective cohort enrolled in our hospital, the expression level of GBP5 was more elevated in CD than in UC. Consistently, the expression levels of GBP5 were correlated with the disease severities of CD, but not that of UC. However, the differential expression of GBP5 was not observed with treatment-naive patients with IBD (Figure 1A). Therefore, the differential expression of GBP5 in CD and UC may be a consequence of different treatments.

In summary, GBP5 is highly expressed in the colonic immune cells of IBD patients. Induction of GBP5 is required for the stimulated production of proinflammatory cytokines and chemokines, while GBP5 deficiency decreases the expression of the proinflammatory mediators. Since GBP5 plays a proinflammatory role at the early steps of the inflammatory cascades of IBD pathogenesis, and is implicated in IBD patients of distinct genetic and environmental backgrounds, targeting GBP5 could be an effective strategy for the management of IBD.

## Materials and Methods

### Human Samples

Colonic pinch biopsies were obtained from patients with IBD at the Sixth Affiliated Hospital of Sun Yat-sen University, Guangzhou, China. Healthy control colonic biopsy samples were from patients suspected of intestinal diseases but diagnosed normal according to biopsy. The simple endoscopic score for Crohn’s disease (SES-CD) and the ulcerative colitis endoscopic index of severity (UC-EIS) were used to evaluate the endoscopic severity of CD and UC, respectively, by endoscopist as previously described (Daperno et al., 2004; Travis et al., 2013). Colon resections were obtained from patients with IBD who underwent colectomy. Non-inflamed control specimens were obtained 5 cm away from the inflamed lesion. Written informed consents were obtained from all donors. This study was approved by the Institutional Review Board of the Sixth Affiliated Hospital of Sun Yat-sen University.

### RNA Extraction, Complementary DNA Synthesis, and Quantitative Real-Time PCR

Total RNA from cultured cells was isolated using TRIzol (Invitrogen, United States). For RNA isolation from clinical samples, colonic tissues were disrupted with lysis beads (Luka, China) before RNA isolation with AllPrep^®^ DNA/RNA Micro Kits (Qiagen, United States). Using total RNA as template, Complementary DNA (cDNA) was synthesized with Fast Reverse Transcription kits (ES Science, China). For the gene expression analysis, qRT-PCR was performed with FastStart Essential DNA Green Master (Roche, United Kingdom) on LightCycler^®^ 96 (Roche, United Kingdom). The gene expression levels were normalized with β-actin gene as the reference gene according to the 2^–ΔΔ*CT*^ method. The sequences of the qRT-PCR primers are as follows: GBP5, forward: 5′-CCTGATGATGAGCTAGAGCCTG-3′, and reverse: 5′-GCACCAGGTTCTTTAGACGAGA; β-actin, forward: 5′-TT GTTACAGGAAGTCCCTTGCC-3′, and reverse: 5′-ATGCT ATCACCTCCCCTGTGTG-3′.

### Transcriptome Analysis

Global transcriptome analysis of the colonic tissue from IBD patients used the published microarray dataset generated from the colonic mucosa of IBD patients and healthy controls (GSE16879)^1^ (Arijs et al., 2009). Data were normalized by MAS5 method before further analysis. Unsupervised hierarchical clustering and Gene Ontology (GO) enrichment were performed with R (3.6.3).

For transcriptome analysis of the wild-type and *GBP5* knockout THP-1 cells, total RNA from parental THP-1 cells and *GBP5* knockout THP-1 clone B2, with and without stimulation (IFNγ and LPS) for 16 h, respectively, was isolated as described above. The RNA preparation was qualified with an Agilent 2100 Bioanalyzer (Thermo Fisher Scientific, United States). Oligo (dT)-attached magnetic beads were used to purify mRNA, which served as templates for cDNA synthesis. Libraries were constructed with the cDNA preparations at BGI-Shenzhen, China, amplified with phi29 to make DNA nanoball (DNB) which had more than 300 copies of one molecular, and sequenced on a BGIseq500 platform (BGI-Shenzhen, China). The sequencing data were filtered with SOAPnuke (v1.5.2) to remove sequences of adapters, to remove reads with low-quality base percentage (base quality less than or equal to 5) higher than 20%, and to remove reads whose unknown base (“N” base) percentage is higher than 5%. The clean reads were mapped to the reference genome using HISAT2 (v2.0.4). After that, EricScript (v0.5.5) and rMATS (V3.2.5) were used to identify fusion genes and differentially spliced genes (DSGs), respectively. Bowtie2 (v2.2.5) was applied to align the clean reads to a human mRNA database built by BGI (Shenzhen, China), and the expression level of gene was calculated by RSEM (v1.2.12). The heatmap was drawn by pheatmap (v1.0.8) according to the gene expression levels. Differential expression analysis was performed using the DESeq2 (v1.4.5) with *Q*-value ≤ 0.05. GO^2^ and KEGG^3^ enrichment analysis of differentially expressed genes was performed by phyper^4^ based on Hypergeometric test. The statistical significance for multiple tests was adjusted by Bonferroni method. Transcriptome data for wild-type and *GBP5* knockout THP-1 cells are available at https://www.biosino.org/node/, accession ID: OEP002938.

### Immunohistochemistry

Formalin-fixed paraffin-embedded intestine tissues from inflamed and non-inflamed sites of IBD patients were sectioned and collected onto glass slides. Antigen retrieval was performed in 10 mM sodium citrate (pH6.0) at 100°C for 15 min. GBP5 was stained with PV-6000 immunohistochemistry (IHC) kit (ZSGB-BIO, China) according to the protocol of the manufacturer, with the GBP5 antibody (Catalog #: 67798) from Cell Signaling (United States). The images were collected with Slide Scanning System SQS-1000 (TEKSQRAY, China). Quantitation of the GBP5 signals was performed with ImageJ with IHC-Toolbox plugins.

### Immunofluorescence

The tissue section was prepared as in IHC method. Primary antibodies include anti-GBP5 (Cat. #: 67798, Cell Signaling, United States), anti-CD3 (Cat. #: 60181-1-Ig, Proteintech, China), anti-CD40 (Cat. #: ab280207, Abcam, United States), and anti-CD68 (Cat. #: ARG10514, Arigo, China). Secondary antibodies include Alexa Fluor647 or 488 conjugated goat anti-rabbit or goat anti-mouse antibodies (Invitrogen, United States). Cell nuclei were stained with 4′,6-diamidine-2′-phenylindole dihydrochloride (DAPI, Sigma-Aldrich, United States). Slides were visualized with a TCS-SP8 confocal microscope (Leica, Germany).

### Cell Culture and Gene Knockdown With siRNA

Human cell lines Jurkat, THP-1, and 293T cells were purchased from American Type Culture Collection (ATCC, United States). Cells were cultured in RPMI 1640 medium (Gibco, United States) supplemented with 10% fetal bovine serum (Gibco, United States) maintained at 37°C and 5% CO2. To knockdown GBP5 in Jurkat cells, a pool of two different siRNAs (1, 5′-GCCATAATCTCTTCATTCA-3′; and 2, 5′-GCTCGGCTTTACTTAAGGA-3′) were used. Scrambled sequence of the siRNA was used as control. RNA was synthesized at Ribo-Bio (China) and provided lyophilized. RNA was reconstituted with nuclease-free water to reach a final concentration of 20μM, before transfection using Lipofectamine RNAiMAX (Life technologies, United States) following the manufacturer’s instructions. At 48 h post-transfection, cells were stimulated with human interferon γ (IFNγ, 25μg/ml, Novoprotein, China) and lipopolysaccharides from Escherichia coli O55:B5 (LPS, 500 ng/ml, Sigma-Aldrich, United States) for 16 h before harvest.

### Western Blot

Cells were lysed in RIPA (Radio-Immunoprecipitation Assay) buffer (P0013B, Beyotime, China) supplemented with protease inhibitors (Cat. #: HY-K0011, MedChemExpress, China) and phosphatase inhibitors (Cat. #: HY-K0021 and HY-K0022, MedChemExpress, China). Lysates were mixed with loading buffer containing SDS and DTT and heated at 95°C for 5 min. Proteins of interest were probed with the following antibodies: anti-GBP5 antibody as described above, anti-GSDMD (Cat. #: 97558, Cell Signaling, United States), anti-caspase 1 (Cat. #: 3866, Cell Signaling, United States), anti-pro-IL1β (Cat. #: 12703, Cell Signaling, United States), and anti-GAPDH (Cat. #: 60004-1-Ig, Proteintech, China). HRP-conjugated secondary antibodies, anti-rabbit IgG and anti-mouse IgG, were from Proteintech, China.

### Guanylate-Binding Protein 5 Knockout Cell Line

Two all-in-one sgRNA plasmids (HCP260159-CG04-3-10-a and HCP260159-CG04-3-10-b) for human *GBP5* were purchased from GeneCopoeia (China). In addition to DNA elements required for the generation of recombinant lentivirus, these plasmids carry Cas9 sequence and one of the following sgRNAs targeting the exon 3 of *GBP5*: gRNA1, 5′-AGTTCTCGATGAGGCACATG-3′; gRNA2, 5′-CATTACGCAACCTGTAGTTG-3′. Recombinant lentivirus was produced by transfecting 293T cells with the two sgRNA plasmids together with lentiviral packaging plasmids psPAX2 and pMD2. *GBP5* knockout THP-1 cells were generated by infecting THP-1 cells with the recombinant lentiviruses followed by G418 selection. After obtaining single-cell colonies from limiting dilution, three THP-1 clones (A1, B1, and B2) harboring all-allelic deletion of the 80-bp in exon 3 of *GBP5* locus were identified by PCR and validated by DNA sequencing of the PCR products. The primers used for PCR screening are as follows: forward, 5′- AGTAGTATGTCCCCAGGTTC-3′, and reverse, 5′- AAGACCAGCTGTAGCCTAAA -3′.

### Luminex-Based Assays

The cell culture supernatant was collected after IFNγ + LPS stimulation. Multiple cytokines and chemokines were examined with Luminex liquid suspension chip at Wayen Biotechnologies Shanghai, Inc. (China). The Bio-Plex Pro Human Chemokine Panel 40-Plex kit (Cat. #: 171AK99MR2, Bio-Rad, United States) was used following the manufacturer’s instructions.

### Statistical Analysis

Data were analyzed by either one-way ANOVA and Dunn’s test or two-tailed Student’s *t*-test using GraphPad Prism (v7.0). Data are represented as the mean ± s.e.m (standard error of the mean). All *in vitro* experiments were performed in triplicate. Correlation analyses were evaluated by Spearman. The number of independent samples and statistical methods used in each experiment is reported in the figure legends. *P* < 0.05 was considered to be statistically significant.

## Data Availability Statement

The datasets presented in this study can be found in online repositories. The names of the repository/repositories and accession number(s) can be found below: https://www.biosino.org/node/, OEP002938; https://www.ncbi.nlm.nih.gov/geo/, GSE16879.

## Ethics Statement

The studies involving human participants were reviewed and approved by the Institutional Review Board of the Sixth Affiliated Hospital of Sun Yat-sen University. The patients/participants provided their written informed consent to participate in this study.

## Author Contributions

LZ and JK conceived and designed the study. XL, YL, SC, YH, YZ, WXW, and JK collected patient samples and clinical data. YL, SC, and YH performed the experiments. YL, XL, LZ, and JK analyzed the data. YL and LZ prepared the manuscript. All authors critically revised the manuscript, had access to the study data, reviewed, and approved the final manuscript.

## Conflict of Interest

The authors declare that the research was conducted in the absence of any commercial or financial relationships that could be construed as a potential conflict of interest.

## Publisher’s Note

All claims expressed in this article are solely those of the authors and do not necessarily represent those of their affiliated organizations, or those of the publisher, the editors and the reviewers. Any product that may be evaluated in this article, or claim that may be made by its manufacturer, is not guaranteed or endorsed by the publisher.

## Footnotes

1
http://www.ncbi.nlm.nih.gov/geo/

2
http://www.geneontology.org/

3
https://www.kegg.jp/

4
https://en.wikipedia.org/wiki/Hypergeometric_distribution

## Abbreviations

AOM: azoxymethane
ASC: apoptosis-associated speck-like protein containing a CARD domain
CD: Crohn’s disease
DSS: dextran sodium sulfate
GBP: guanylate-binding protein
GSEA: gene set enrichment analysis
IBD: inflammatory bowel diseases
IFN: interferon
IHC: immunohistochemistry
LPS: lipopolysaccharide
NLRP3: NOD-like receptor family pyrin domain containing 3
PBMCs: peripheral blood mononuclear cells
SES-CD: simple endoscopic score for Crohn’s disease
SNP: single-nucleotide polymorphism
TNBS: 2,4,6-trinitrobenzene sulfonic acid
UC: ulcerative colitis
UC-EIS: ulcerative colitis endoscopic index of severity.
