# Supplementary material for: The Proinflammatory Role of Guanylate-Binding Protein 5 in Inflammatory Bowel Diseases
Source: Front Microbiol. 2022 Jun 2;13:926915. doi: 10.3389/fmicb.2022.926915 (PMC9201962; doi:10.3389/fmicb.2022.926915)
Supplement: Supplementary file 1 [file Data_Sheet_1.docx]

Supplementary Material

# Supplementary Figures and Tables

##
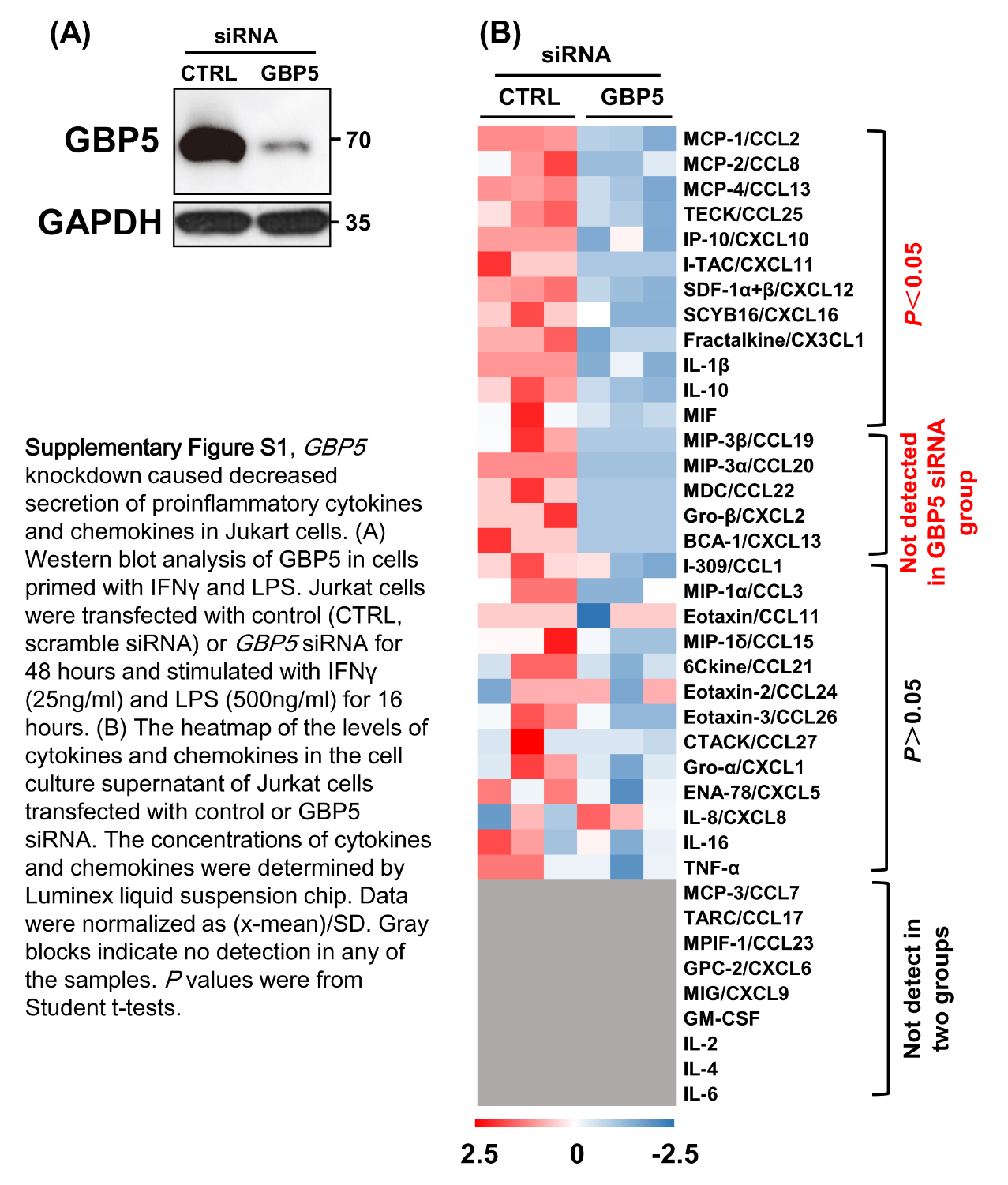
Supplementary Figures


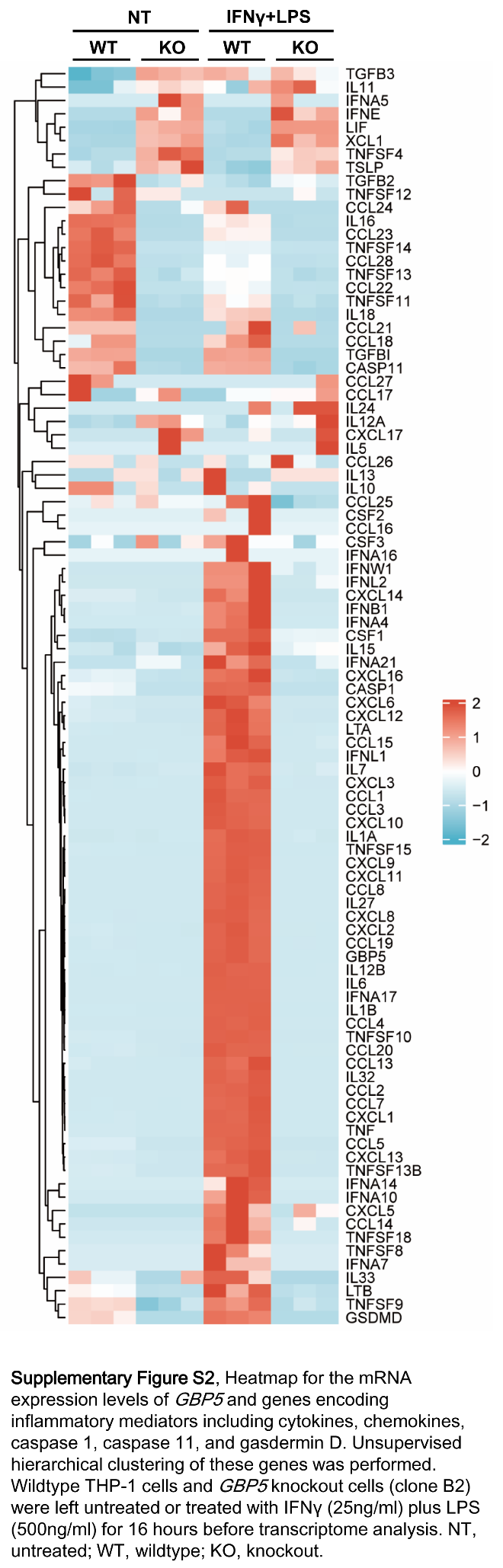


## Supplementary Table

| Supplementary Table S1, Clinical characteristics of IBD patients and control subjects | | | |
| --- | --- | --- | --- |
|  |  |  |  |
| Characteristic | Control (*n*=35) | CD (*n*=37) | UC (*n*=18) |
| Male/female (%) | 21/14 (60/40) | 25/12 (68/32) | 9/9 (50/50) |
| BMI | 24 (21-26) | 18 (17-20) | 22 (18-24) |
| Median (IQR) age at sampling (years) | 46 (39-54) | 27 (19-34) | 40 (27-54) |
| Median (IQR) age at diagnosis (years) | n/a | 23 (18-29) | 35 (23-48) |
| Median (IQR) disease duration (years) | n/a | 3 (1.5-6.5) | 5 (1-8) |
| Median (IQR) C-reaction protein (mg/l) | 0.7 (0.4-1.8) | 13.1 (1.3-39.9) | 0.8 (0.5-7.0) |
| Median (IQR) peripheral blood leukocytes (10^9/l) | 5.7 (5.0-7.0) | 5.8 (4.7-7.4) | 6.3 (4.9-8.1) |
| Current medication at sampling (%) |  |  |  |
| 5-Aminosalicylates | 0 | 11 (30) | 15 (83) |
| Corticosteroids | 0 | 7 (19) | 4 (22) |
| Purine pathway inhibitors (e.g. azathioprine) | 0 | 13 (35) | 0 |
| Infliximab | 0 | 16 (43) | 1 (6) |
| Methothexate | 0 | 3 (8) | 0 |
| Antibiotic | 0 | 6 (16) | 4 (22) |
| Thalidomide | 0 | 6 (16) | 0 |
| Monotherapy only (one of the above) | 0 | 13 (35) | 9 (50) |
| Combination of two or more of the above | 0 | 16 (43) | 7 (39) |
| No treatment | 35 (100) | 3 (8) | 2 (11) |
| Unkown | 0 | 3 (8) | 0 |
|  |  |  |  |
| Data are median (IQR) or n (%). BMI, body mass index; IQR, interquartile range; n/a, not applicable. | | | |
